# Indoor and Outdoor Monitoring of Volatile Organic Compounds in School Buildings: Indicators Based on Health Risk Assessment to Single out Critical Issues

**DOI:** 10.3390/ijerph10126273

**Published:** 2013-11-25

**Authors:** Gianluigi de Gennaro, Genoveffa Farella, Annalisa Marzocca, Antonio Mazzone, Maria Tutino

**Affiliations:** Department of Chemistry, University of Bari, via Orabona, 4, Bari 70126, Italy; E-Mails: jenny_f89@hotmail.it (G.F.); annalisa.marzocca@uniba.it (A.M.); antonio.mazzone@uniba.it (A.M.); maria.tutino@uniba.it (M.T.)

**Keywords:** indoor air quality, volatile organic compounds, indoor/outdoor plot, indicators based on health risk assessment

## Abstract

Children are more sensitive to pollutants than adults and yet they spend large amounts of time in school environments where they are exposed to unknown levels of indoor pollutants. This study investigated the concentrations of the most abundant volatile organic compounds (VOCs) in eight naturally ventilated school buildings in Italy. The schools were chosen to include areas with different urbanization and traffic density characteristics in order to gather a more diverse picture of exposure risks in the different areas of the city. VOCs were sampled for one week in the presence/absence of pupils using diffusive samplers suitable for thermal desorption inside three classrooms at each school. The samples were then analyzed with thermal desorption-gas chromatography-mass spectrometry (TD-GC-MS). In addition, outdoor measurements were carried out in the yard at each school. VOC identification and quantification, and indoor/outdoor concentration plots were used to identify pollutant sources. While some classrooms were found to have very low VOC levels, others had a significant indoor contribution or a prevalent outdoor contribution. High concentrations of terpenes were found in all monitored classrooms: α-pinene and limonene were in the range of 6.55–34.18 µg/m^3^ and 11.11–25.42 µg/m^3^ respectively. Outdoor concentrations were lower than indoors for each monitored school. Indicators based on health risk assessment for chronic health effects associated with VOCs (either carcinogenic or non-carcinogenic) were proposed to rank sites according to their hazard level.

## 1. Introduction

Children are more sensitive to pollutants than adults and yet they spend large amounts of time in school environments where they are exposed to unknown levels of indoor pollutants [1,2,3,4,5]. Several studies have reported that indoor air pollution can increase the likelihood of long-term and short-term health problems for students and teachers creating a situation which may affect comfort, productivity and academic performance [2,6,7,8,9,10,11]. Despite the large number of school-aged children and their sensitivity to indoor pollutants, information on this topic has been limited [6,12]. Sources of indoor pollution in school buildings can be traced back to a variety of causes such as the use of high emitting materials for building construction and furnishing, minimal landscaping with poor drainage, the type of heating, ventilation and air conditioning units (Heating, Ventilation, and Air Conditioning—HVAC), the lack of preventative maintenance, crowded conditions and cleaning products that release chemicals into the air [12,13]. Other factors influencing the concentration of pollutants are the age and location of a school building, the presence of pollutants from outdoor sources, chemical reactions in indoor air and heterogeneous processes at air-solid interfaces [14]. Since each school environment is unique, the levels of both outdoor and indoor pollutants need to be considered to determine each individual’s exposure level.

In general, chronic health effects provoked by VOCs can be classified as either non-carcinogenic or carcinogenic. Some VOCs may be associated with a variety of serious health effects and symptoms such as asthma and allergic reactions [7,8,9,10,11,12,13,14,15]. Moreover, several studies on office workers have reported a strong association between problems such as mucous membrane irritation and central nervous system symptoms, and the total exposure to VOCs. These symptoms are also very similar to those frequently attributed to Sick Building Syndrome (SBS) [16,17]. The main carcinogenic effects are lung, blood (leukemia and non-Hodgkin lymphoma), liver, kidney and biliary tract cancer. The International Agency for Research on Cancer (IARC) has classified benzene as a human carcinogen (Group 1), while other VOCs such as tetrachloroethylene and ethylbenzene are considered to be known or probable carcinogens for humans (Group 2A or 2B, IARC). In developed countries, many studies have been conducted during the past decade in order to assess IAQ in school environments and a large number of indoor air pollutants have been measured including carbon dioxide (CO_2_), ozone (O_3_), nitrogen oxides (NO_x_), carbon monoxide (CO), sulphur dioxide (SO_2_), volatile organic compounds (VOCs), and particulate matter (PM) [6,7,10,11,12,18,19,20,21,22,23]. Several studies worldwide have focused on the assessment of pupils’ exposure to PM in classrooms, but few authors have yet to report results on PM chemical composition and size [23,24,25,26,27,28,29].

The aim of the present study is to characterize IAQ in eight primary school buildings located in different areas of Bari in southeast Italy. The study carried out simultaneous indoor and outdoor VOC concentration measurements and assessed the influence of outdoor emissions and weather on the IAQ of the naturally ventilated classrooms investigated. Moreover, two integrated indicators were introduced to correlate reference values used to describe chronic health effects (carcinogenic and non-carcinogenic) with the VOC concentrations found in these environments. The monitored VOCs included many different chemical classes like aliphatic hydrocarbons such as alkanes and cycloalkanes, aromatic hydrocarbons, terpenes, aldehydes, ketones, alcohols and halocarbons. This variety of compounds have been associated with health effects ranging from those with no known effect, produced by rather inert VOCs, to those with a highly toxic effect, produced by reactive VOCs.

## 2. Experimental Section

Indoor air quality parameters were investigated in eight naturally ventilated school buildings. Three classrooms were chosen for each school so that they were similar for characteristics such as level in the school building, surface, volume, number of windows, windows structure, number of occupants, activities, internal covering including flooring, wall and ceiling. Furthermore parallel outdoor measurements were carried out in the yard of each school. The classrooms were occupied during class time on school days for a total of 30 h during the school week. They were unoccupied during afternoons, evenings and nights on school days for a total of about 90 h during the week. Class started at 08:00 and finished at 14:00. Three samplers were exposed in each monitored environments: one in the presence of pupils, one in the absence of pupils and one for the entire school week (from Monday to Friday for a total of about 120 h). The samplers were positioned at a height of about 1.5 m above the floor and at a distance that exceeded 1m from any window or door. Instead, outdoor VOC measurements were collected weekly (one outdoor sampler for each school) at heights of about 2 m above the ground [30,31].

### 2.1. Sampling Sites

Table 1 lists the main characteristics of the eight monitored schools.

**Table 1:** Characteristics of monitored sites.

Monitored sites	Kind of school	Description
School 1	Middle school	Located in a central area
		Surrounded by residential and commercial buildings
		Proximity to a trafficated road
		Use of interactive whiteboards
School 2	Middle school	Located in a suburban area
		Surrounded by residential and buildings
		Use of blackboard with chalk
School 3	Middle school	Located in a suburban area
		Surrounded by residential buildings and fallow fields
		Presence of a garden
		Use of blackboard with chalk
School 4	Middle school	Located in a suburban area
		Surrounded by residential buildings
		Presence of a garden
		Use of blackboard with chalk
School 5	Elementary school	Located in a central area
		Surrounded by residential and commercial buildings
		Proximity to a trafficated road
		Use of interactive whiteboards
School 6	Elementary school	Located in a central area
		Surrounded by residential and commercial buildings
		Proximity to a trafficated road
		Use of blackboard with chalk
School 7	Elementary school	Located in a suburban area
		Surrounded by residential and commercial buildings
		Use of blackboard with chalk
School 8	Elementary school	Located in a suburban area
		Surrounded by residential and commercial buildings
		Use of blackboard with chalk

### 2.2. Sampling and Analytical Method

VOCs were sampled with Radiello^®^ diffusive samplers (Fondazione Salvatore Maugeri, Padova, Italy) suitable for thermal desorption. The sampling system was made up of a cylindrical adsorbing cartridge housed coaxially inside a cylindrical diffusive body of polycarbonate and microporous polyethylene. Each cartridge consisted of a cylindrical adsorbing cartridge which was a stainless steel net cylinder with 100 mesh. It had an external diameter of 4.8 mm, containing 350 mg of Carbograph 4 (35–50 mesh). Before sampling, the cartridges were conditioned and analysed to verify blank levels [32,33]. Each sampler was exposed for the periods indicates in the experimental design and then closed in a sealed glass tube and brought to the laboratory for analysis. The analyses were carried out using a thermal desorber (Markes International Ltd., Unity™, Llantrisant, UK) equipped with an autosampler (Markes mod. ULTRA™ TD) which was provided with 100 positions and coupled with a gas chromatograph (Agilent GC-6890 PLUS, Loveland, Colorado, USA) and a mass selective detector (Agilent MS-5973N). The thermal desorber had a two-stage mechanism: first the analytes were desorbed from the sample tube and refocused into a cold trap; then they were desorbed from the trap and carried into the GC column [32,33,34]. Standard solutions were prepared by injecting successive dilution in methanol of a VOC standard mixture at 2,000 µg/mL (Cus-5997 Ultra Scientific, Bologna, Italy). To quantify the samples, the calibration curves were prepared by injecting 1 µL of the standard solution into a tube; the spiked adsorbent tubes were then thermally desorbed in the same conditions of time, gas flow and split ratio as the samples. The sampling rates, Q values supplied by manufacturer, were useful to calculate the real concentration of compound in the atmosphere (C) by GC quantification of analytes mass, m. Q was a function of the diffusive coefficient D, which was the thermodynamic property of each chemical substance. The sampling rate had the dimensions of a gaseous flow: when m was expressed in µg, the sampling period in minutes and C in µg/L, Q was expressed in L/min [35]. The assessment of the performance and reliability of the indoor monitoring methodology to determine VOC concentrations using radial diffusive samplers for thermal desorption was presented in previous works [32,33]. In particular the repeatability of the analysis for thermal desorption, the complete desorption of the cartridges, the limit of detection (LOD), and the limit of quantification (LOQ) were evaluated. The results showed that the RSD% was less than 10 for all compounds. The percentage recovery was higher than 95%, confirming the high method reliability for VOC analysis.

### 2.3. Integrated Indicators for IAQ Based on Health Risk Assessment

In this study, two integrated indicators based on inhalation risk assessment were proposed in order to gain an overall assessment of indoor air quality (IAQ) in the monitored environments. In particular, the indicators were obtained by considering the reference values used to describe chronic health effects (carcinogenic and non-carcinogenic) of VOCs: the IAQ Cancer Risk Indicator (CRI) which concerns cancer risk and the IAQ Total Hazard Ratio Indicator (THRI) relating to non-cancer risk.

#### 2.3.1. IAQ Cancer Risk Indicator (CRI)

IAQ Cancer Risk Indicator was created using the available Unit Risk (UR) estimated values that reflect a dose which corresponds to a specific level of cancer risk. In particular, the UR is defined as the upper-bound excess lifetime cancer risk estimated to result from continuous exposure to an agent at a concentration of 1 µg/m^3^ in air [36]. The inhalation UR values for the carcinogenic VOCs detected in this study were extracted from the database provided by the Integrated Risk Information System (IRIS) and the California Environmental Protection Agency (CalEPA) (see Table 2).

**Table 2:** Cancer unit risks of the VOCs found during the monitoring campaign.

Compound	UNIT RISK (µg/m^3^)	SOURCE
Benzene	7.80*10-6	IRIS
Ethyl-benzene	2.50*10-6	CALEPA
1,4-Dichlorobenzene	1.10*10-5	CALEPA
Tetrachloroethylene	2.60*10-7	IRIS

IRIS (Integrated Risk Information System) [36]; CalEPA (California Environmental Protection Agency (OEHHA) Office of Environmental Health Hazard Assessment’s) [37].

The lifetime cancer risk (LCR) attributable to inhalation exposures was calculated by multiplying UR estimated value by the ambient concentration (μg/m^3^):

LCR = C × UR
(1)

Since the individual cancer risk of each VOC was additive, the global lifetime cancer risk was calculated as the sum of the LCR of each compound. Hence, to define a comprehensive LCR for each site, the global LCR (LCR_site_) was determined by summing each specific LCR_i_ for all the chemicals found at each site:

LCR_site_ = ∑LCR_i_ (2)

LCR_site_ values were used to obtain the IAQ Cancer Risk Indicators.

#### 2.3.2. IAQ Total Hazard Ratio Indicator (THRI)

IAQ Total Hazard Ratio Indicator (THRI) was based on the comparison of the daily ambient concentrations with their respective chronic non-cancer inhalation level (reference concentrations), the point at which no adverse effects are expected for a single VOC. Non-cancer reference concentrations of VOCs detected in this study were extracted from the database provided by the IRIS and the Provisional Peer Reviewed Toxicity Values for Superfund (PPRTV) [36,37,38] (see Table 3).

**Table 3:** Non-cancer reference concentrations (µg/m^3^) of the VOCs found during the monitoring campaign.

Compound	REFERENCE CONCENTRATION (µg/m^3^)	SOURCE
Benzene	30	IRIS
Toluene	5,000	IRIS
Tetrachloroethylene	40	IRIS
Ethyl-benzene	1,000	IRIS
m-Xylene	100	IRIS
Styrene	1,000	IRIS
1,4-Dichlorobenzene	800	IRIS
1,2,3-Trimethylbenzene	5,000	PPRTV

IRIS (Integrated Risk Information System) [36]; PPRTV (Provisional Peer Reviewed Toxicity Values of IRIS) [38].

The hazard ratio (HR) of each compound (i) was calculated by dividing its average concentration by its corresponding reference concentration (RfC), both expressed in μg/m^3^:

HR_i_ = C_i_/RfC_i_ (3)

Moreover, the total hazard ratio (THR_site_) was calculated to assess the global inhalation exposure risk, for each monitored classroom, as a sum of the single HR_i_ determined for each compound checked at the same sample site:

THR_site_ = ∑ HR_i_ (4)

## 3. Results and Discussion

The VOC concentrations measured at all the sites are listed in Table 4. VOCs concentrations detected in this study were above or at most on line with those in other studies [12,13,14,15]. α-Pinene, camphene and limonene were the most abundant compounds in most of the schools. Critical issues were detected in some schools such as high levels of 1,2,3-trimethylbenzene in schools 5 and 8, and of *m*,*p*-xylene in schools 3 and 6. Although the monitored schools were located in different areas of the city, characterized by different density of vehicular traffic, similar and low outdoor concentrations were detected. This findings was probably due to the fact that the outdoor sampler were exposed in the yard of each school (according to UNI EN ISO 16000-1) so that detected concentrations were representative of the air in proximity of the indoor site. Therefore, outdoor sites probably were less affected by vehicular traffic emissions.

**Figure 1:**
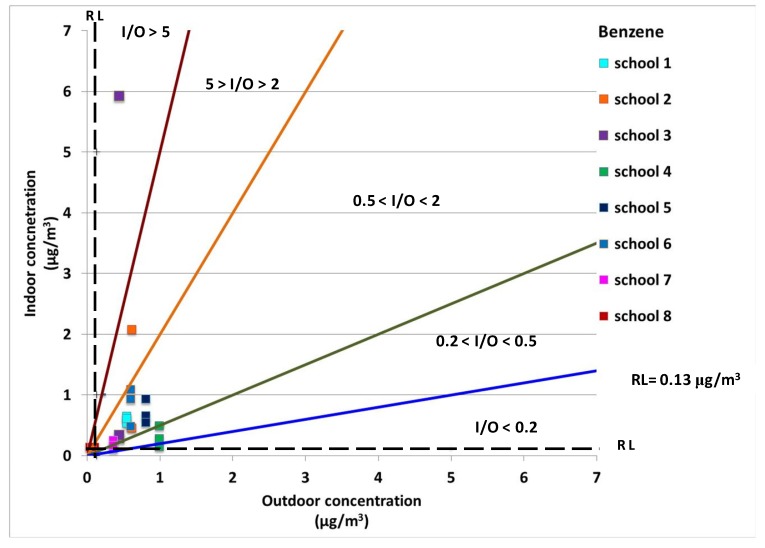
Benzene indoor concentrations against outdoor concentrations in all monitored sites. The reference level (RL) and five different I/O ranges were also displayed.

Indoor/outdoor (I/O) ratios were also calculated for each classroom in order to understand whether or not VOC sources were located indoors or outdoors (Table 5). I/O ratios are commonly used to highlight the presence of important indoor emission sources. Ratios greater than a defined threshold value indicated the predominance of indoor contributions over an outdoor one. Different threshold values have been used in previous works [32,33]. In this study I/O ratios were used to single out critical issues that may have originated from significant indoor sources (if I/O is very high) or from outdoor ones (if I/O < 1). The initial data analysis showed that indoor concentrations of benzene and substituted benzenes are due to the input/intrusion of VOCs from outdoor areas in most of the classrooms. Indeed, benzene and substituted benzenes are known as markers of vehicular traffic emissions [39,40]. Nevertheless, these situations had still to be related to the concentration levels since two different sites having the same I/O ratio could actually have issues originating from a different source. For this purpose, the indoor concentration value for each pollutant was plotted against the corresponding outdoor value and five different I/O ranges were defined (see Figure 1).

**Figure 2:**
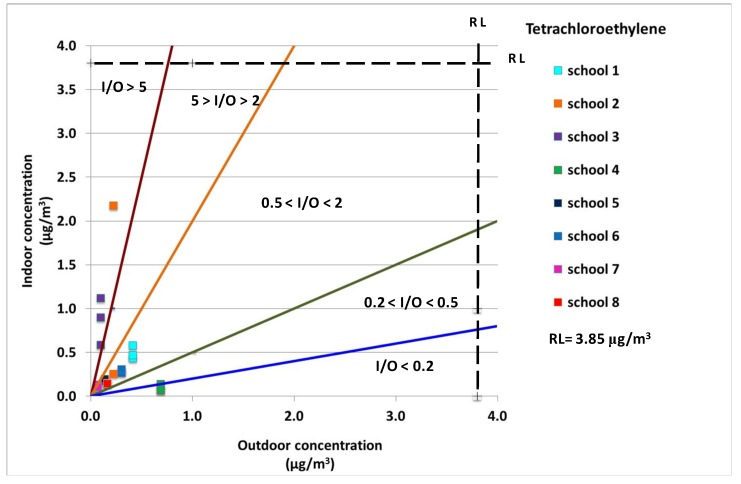
Tetrachloroethylene indoor concentrations against outdoor concentrations in all monitored sites. The reference level (RL) and five different I/O ranges were also displayed.

**Figure 3:**
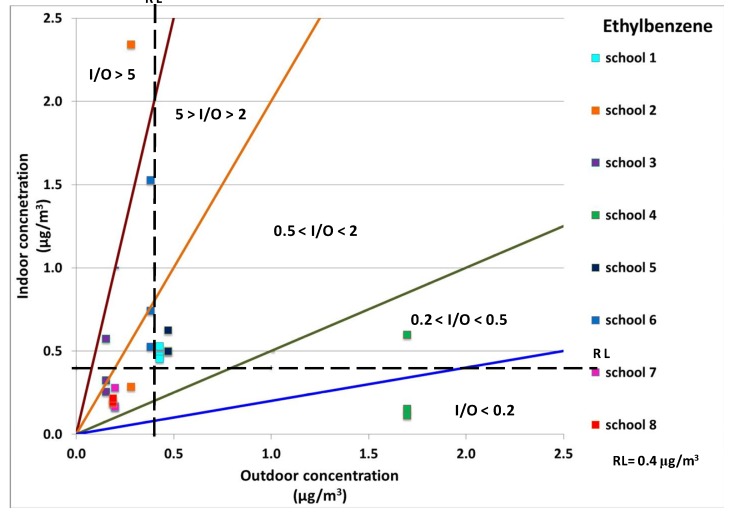
Ethylbenzene indoor concentrations against outdoor concentrations in all monitored sites. The reference level (RL) and five different I/O ranges were also displayed.

**Figure 4:**
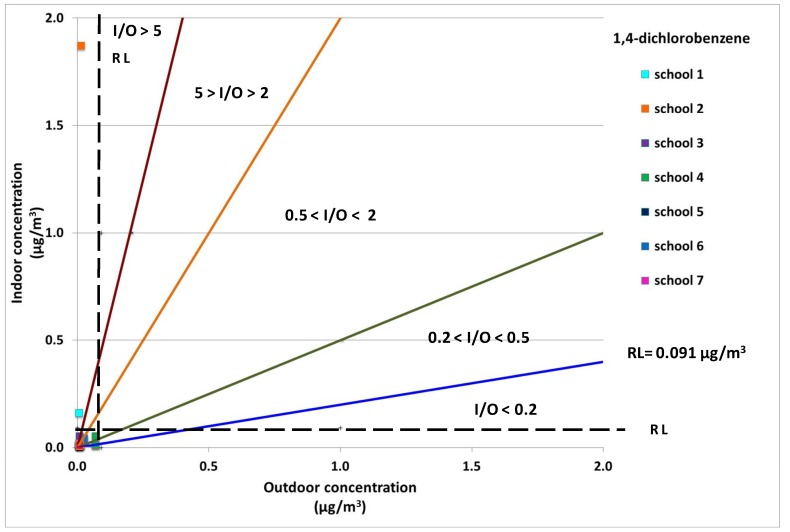
1,4-Dichlorobenzene indoor concentrations against outdoor concentrations in all monitored sites. The reference level (RL) and like different I/O ranges were also displayed.

Figure 1, Figure 2, Figure 3 and Figure 4 show the indoor concentrations against outdoors for the cancer compounds monitored in this study: benzene, ethylbenzene, 1,4-dichlorobenzene and tethrachloroethylene. Moreover, the reference level (RL), which is the concentration level for each compound where excess cancer cases are expected to develop per 1,000,000 people if exposed daily for a lifetime, is given. Figure 1 shows that school 2 (I/O > 5) and school 3 (5 > I/O > 2) presented two different yet critical indoor air quality situations. School 2 (classroom 2) and school 3 (classroom 1) had indoor benzene concentrations 13 and 3 times higher than the outdoor concentrations, respectively (see Table 5). This finding suggests that there were high indoor sources of benzene in these classrooms. Furthermore, similar results were found for tetrachloroethylene (see Figure 2). The indoor concentrations of tetrachloroethylene in school 2 (classroom 2) and school 3 (all classrooms) were 9, 8, 5, and 11 times higher than the outdoor concentrations, respectively. However, the tetrachloroethylene concentrations were generally very low in schools 2 and 3, with a maximum value of 2.2 μg/m^3^ and 1.1 μg/m^3^, respectively (see Table 4).

Indoor and outdoor concentrations of ethylbenzene are plotted in Figure 3. Schools 2 and 3 had the highest values for this pollutant, but also school 6 (classroom 2) had indoor concentrations 4 times higher than those outdoors. The concentration levels of 1,4-dichlorobenzene were very low at all the sites. Only school 2 showed indoor concentrations that were about 2 times higher than the outdoor ones (see Figure 4). These findings clearly demonstrated that the values of the I/O ratios were insufficient for highlighting critical issues especially when values ranged from 0.5 to 2.

**Table 4:** VOC concentrations at monitored sites.

Compounds	Concentration µg/m^3^																							
	School 1			School 2			School 3			School 4			School 5			School 6			School 7			School 8		
	Indoor		Outdoor	Indoor		Outdoor	Indoor		Outdoor	Indoor		Outdoor	Indoor		Outdoor	Indoor		Outdoor	Indoor		Outdoor	Indoor		Outdoor
	Min	Max		Min	Max		Min	Max		Min	Max		Min	Max		Min	Max		Min	Max		Min	Max	
**Benzene**	0.53	0.65	0.55	0.45	2.07	0.62	0.29	5.93	0.44	0.15	0.49	1.00	0.55	0.65	0.81	0.47	1.09	0.60	0.11	0.25	0.37	0.04	0.11	0.14
**Heptane**	0.47	1.46	0.14	0.86	4.58	0.07	0.21	3.22	0.02	0.11	0.17	0.42	0.33	3.13	0.16	0.38	5.36	0.10	0.32	0.89	0.09	0.05	0.06	0.10
**Toluene**	1.88	2.26	2.06	1.16	3.70	1.29	1.08	1.72	0.73	1.33	1.54	5.62	2.55	4.84	2.47	4.12	6.81	1.63	0.73	0.97	0.73	0.83	0.86	0.83
**Tetrachloroethylene**	0.43	0.58	0.41	0.25	2.17	0.23	0.58	1.11	0.10	0.07	0.13	0.69	0.19	0.19	0.14	0.26	0.31	0.31	0.09	0.13	0.07	0.14	0.15	0.17
**n-Butylacetate**	0.55	0.86	0.52	0.32	2.60	0.18	0.36	0.39	0.12	0.26	0.39	2.23	0.85	1.08	0.98	0.48	0.70	0.59	0.19	0.29	0.11	0.19	1.01	0.12
**Ethyl-benzene**	0.45	0.53	0.43	0.28	2.34	0.28	0.26	0.57	0.15	0.11	0.60	1.70	0.50	0.62	0.47	0.53	1.53	0.38	0.16	0.28	0.20	0.19	0.22	0.19
**m,p-Xylene**	1.34	1.59	1.46	0.84	2.82	0.89	0.60	21.03	0.41	0.25	1.05	2.38	1.58	2.01	1.65	1.66	5.84	1.13	0.48	0.82	0.57	0.56	0.59	0.60
**Styrene**	0.62	0.83	0.45	0.22	2.26	0.21	<0.05 *	0.24	0.14	0.18	0.39	1.26	0.27	0.35	0.18	0.32	0.59	0.26	0.12	0.24	0.16	0.12	0.14	0.12
**Alpha-pinene**	6.55	34.18	<0.03 *	1.67	8.26	2.89	0.85	1.87	0.27	1.38	8.60	3.08	0.92	1.21	0.23	0.56	5.14	<0.03 *	1.13	4.05	0.34	0.37	2.29	0.72
**Camphene **	2.26	3.05	15.22	1.30	9.17	0.51	0.54	0.85	0.29	1.89	4.42	3.62	0.92	3.01	0.12	1.06	2.67	0.04	8.99	11.03	0.68	0.79	1.85	1.32
**Decane**	2.10	3.83	2.81	0.82	1.63	1.61	1.13	3.69	0.52	1.32	5.86	4.74	3.25	4.04	0.38	1.28	1.92	0.38	0.40	2.91	0.43	0.28	0.90	0.50
**1,4-Dichlorobenzene**	0.02	0.16	0.01	0.01	1.87	0.02	0.01	0.05	0.01	0.01	0.05	0.07	0.01	0.01	0.01	0.04	0.04	0.03	0.01	0.02	0.01	0.01	0.01	0.01
**1,2,3-Trimethylbenzene**	0.38	0.45	0.34	0.33	0.99	0.30	0.18	0.47	0.13	0.28	0.46	0.76	0.39	61.00	0.27	0.37	0.86	0.17	0.11	0.18	0.18	0.14	19.00	0.14
**Limonene**	11.11	25.42	32.15	9.42	10.73	10.25	3.67	4.63	1.03	6.08	8.60	10.51	2.51	4.01	0.36	2.24	4.79	0.08	4.21	6.10	1.22	1.30	2.62	1.01

* LOD.

**Table 5:** I/O ratios for each monitored classroom.

Compounds	School 1			School 2		School 3			School 4			School 5			School 6			School 7			School 8		
	classroom 1	classroom 2	classroom 3	classroom 1	classroom 2	classroom 1	classroom 2	classroom 3	classroom 1	classroom 2	classroom 3	classroom 1	classroom 2	classroom 3	classroom 1	classroom 2	classroom 3	classroom 1	classroom 2	classroom 3	classroom 1	classroom 2	classroom 3
**Benzene**	1.0	1.2	1.1	0.7	3.4	13.4	0.6	0.8	0.3	0.5	0.1	0.8	0.8	0.7	1.5	0.8	1.8	0.3	0.7	0.5	0.8	0.3	0.5
**Heptane**	8.4	3.4	10.7	12.9	69.1	157.4	83.3	10.2	0.4	0.3	0.3	3.4	20.0	2.1	53.9	4.2	3.8	3.6	9.9	4.5	0.6	0.5	0.6
**Toluene**	1.0	0.9	1.1	0.9	2.9	2.4	1.5	1.5	0.2	0.3	0.2	2.0	1.3	1.0	4.2	2.5	3.3	1.3	1.3	1.0	1.0	1.0	1.0
**Tetrachloroethylene**	1.4	1.0	1.1	1.1	9.6	8.9	5.8	11.0	0.1	0.2	0.1	1.4	1.4	1.4	0.9	0.9	1.0	1.3	1.8	1.4	0.9	0.9	0.8
**n-Butylacetate**	1.1	1.2	1.7	1.8	14.4	3.1	3.4	3.3	0.1	0.1	0.2	1.1	0.9	1.1	0.8	1.2	0.9	1.7	2.7	2.0	1.6	1.9	8.6
**Ethyl-benzene**	1.1	1.2	1.1	1.0	8.3	3.7	2.1	1.6	0.1	0.4	0.1	1.1	1.3	1.1	1.9	4.0	1.4	0.8	1.4	0.8	1.0	1.0	1.1
**m-Xylene**	1.0	1.1	0.9	0.9	3.2	51.7	1.5	1.5	0.2	0.4	0.1	1.0	1.2	1.0	1.8	5.2	1.5	0.9	1.4	0.8	0.9	0.9	1.0
**Styrene**	1.4	1.4	1.8	1.0	10.8	n.d	1.7	1.7	0.1	0.3	0.1	1.9	1.7	1.5	1.6	2.2	1.2	0.7	1.5	0.8	0.9	1.2	1.0
**Alpha-pinene**	n.d	n.d	n.d	0.6	2.9	3.2	7.0	4.1	2.8	0.8	0.4	4.7	4.0	5.2	n.d	n.d	n.d	n.d	11.8	3.3	1.6	3.2	0.5
**Camphene **	0.2	0.2	0.1	2.5	17.9	3.0	1.9	2.2	0.5	0.6	1.2	24.5	7.5	14.4	64.1	27.1	25.5	16.1	13.1	13.5	1.4	0.6	1.2
**Decane**	1.3	1.4	0.7	1.0	0.5	7.1	4.2	2.2	1.2	0.5	0.3	10.6	8.5	9.0	4.8	5.1	3.4	0.9	1.3	6.8	1.1	1.8	0.6
**1,4-Dichlorobenzene**	2.0	1.8	19.0	0.7	120.7	5.8	1.3	0.9	0.1	0.7	0.1	1.1	0.9	1.2	1.4	1.6	1.7	0.6	1.7	0.6	1.1	2.3	1.4
**1,2,3-Trimethylbenzene**	1.2	1.1	1.3	1.1	3.3	3.7	1.6	1.4	0.4	0.6	0.2	1.6	2.2	1.4	3.4	5.2	2.2	0.6	1.0	0.8	1.1	1.4	1.0
**Limonene**	0.8	0.5	0.3	1.0	0.9	4.5	4.0	3.6	0.7	0.6	0.8	11.2	10.4	7.0	63.3	29.6	36.5	5.0	3.4	3.6	1.3	2.6	1.3

n.d: not detectable.

The CRI for each indoor and outdoor site was calculated for the VOCs that exhibit carcinogenic activity: benzene, ethylbenzene, 1,4-dichlorobenzene, and tetrachloroethylene (see UR values in Table 2). The individual cancer risk of each VOC was assumed to be additive and the CRI was calculated as the sum of the LCRi of the individual compounds [41].

**Figure 5:**
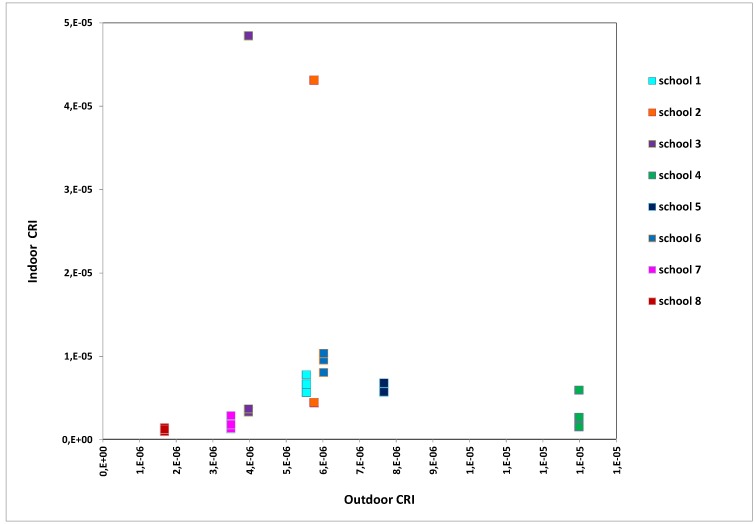
Indoor CRI against outdoor CRI for each monitored school.

**Figure 6:**
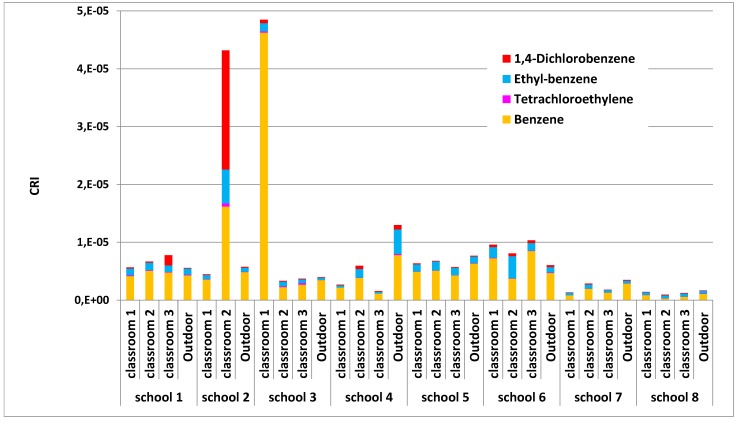
Cancer Risk Indicator in each monitored site.

The indoor CRI were plotted against the outdoor ones for each monitored school (see Figure 5) confirming that school 2 and school 3 were environments with a high level of concern. Figure 6 shows that school 2 (classroom 2) and school 3 (classroom 1) presented the highest CRI values among the monitored environments (see Figure 6). On the other hand, schools 7 and 8 were found to be the healthiest environments. School 4, despite having high CRI values outside, had good indoor air quality, whereas the IAQ of school 5 was affected by its outdoor concentrations. Finally, schools 1 and 6 had indoor CRIs equal to or slightly greater than those outdoors.

**Figure 7:**
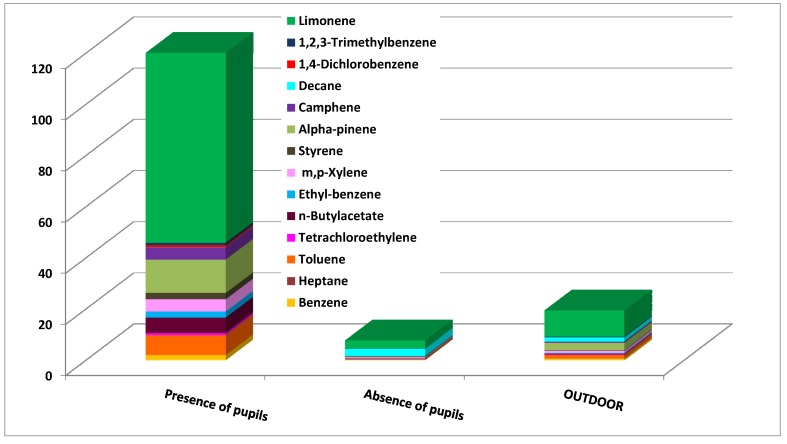
VOC concentration in classroom 2, school 2 in presence and in absence of pupils.

**Figure 8:**
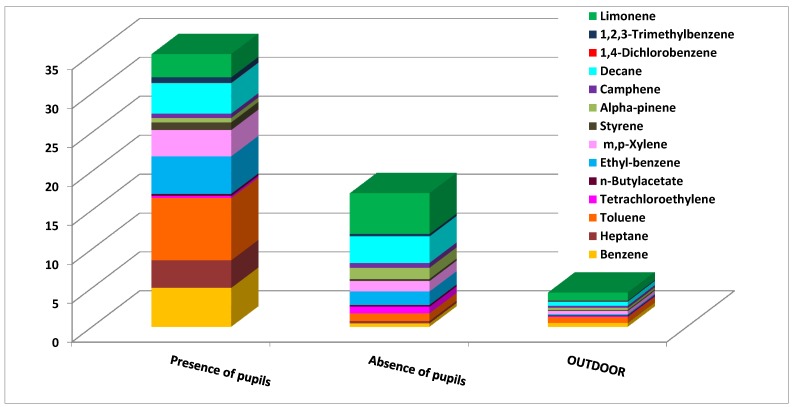
VOC concentration in classroom 1, school 3, in presence and in absence of pupils.

Benzene was found to be the highest single compound contributor to the CRI values in all the schools. The comparison of indoor and outdoor contributions showed immediately that, in most environments, the indoor benzene concentrations were similar or slightly higher than those outside. This finding suggests that the indoor concentrations may have primarily been due to outdoor emission sources. Schools 2 and 3 deserve particular attention since here the indoor contributions of benzene to the CRI were much higher than the outdoor ones. High contributions of 1,4-dichlorobenzene and ethylbenzene were also obtained in school 2 (classroom 2). Critical concentrations were detected in school 2 (classroom 2) (Figure 7) and school 3 (classroom 1) (Figure 8). These situations were then intensely studied by comparing the indoor concentrations of all of the monitored VOCs during the presence and absence of pupils. Figure 7 illustrates that the VOC concentrations were higher when pupils were present. This finding suggests that activities during school hours may have produced the detected VOC levels. In particular, it was found that limonene, α-pinene and toluene were the most abundant pollutants.

Limonene and α-pinene are odorous compounds mainly used to give pleasant fragrance and flavoring to school supplies, food, cosmetics, personal care products, and cleaning products [42,43]. Furthermore, the high concentration of toluene during student activities confirmed the presence of indoor sources in this school. The presence of this particular compound may have originated from solvent-based paints, adhesives, PVC flooring, carpeting, printed material and solvent-based consumer products [44]. The high contribution of toluene detected in school 3 confirmed that this pollutant is strictly connected to the pupils’ activities. In school 3, higher contributions of heptane, ethylbenzene, and *m*,*p*-xylene were also detected during school hours while the decane contribution was high during both the presence and absence of pupils. This suggests that the materials or furniture present in classroom 1 may be the main emission sources of decane.

**Figure 9:**
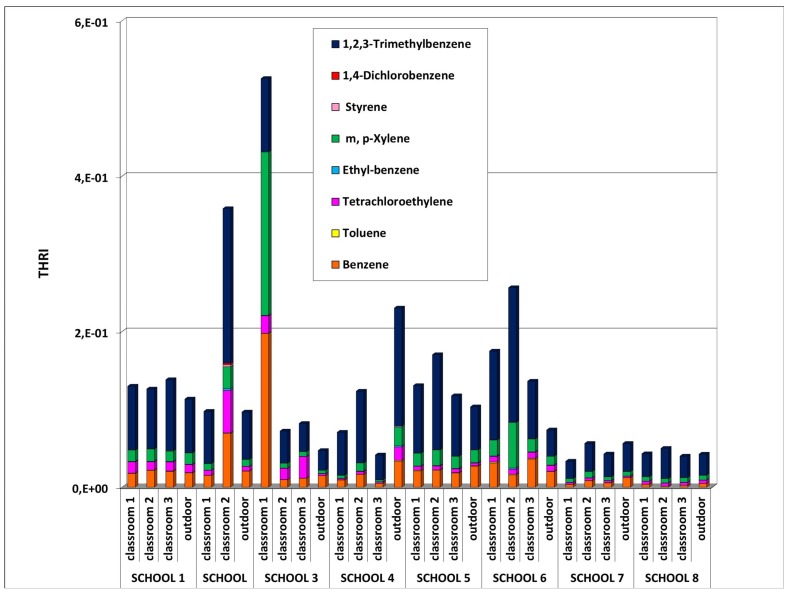
THRI values for each monitored site.

The second indicator was the IAQ Total Hazard Ratio Indicator (THRI) based on the non-carcinogenic risk assessment. This was calculated by considering the largest possible number of detected compounds. Among the investigated compounds, reference values for non-cancer effects were estimated for eight quantified VOCs (see Table 3). THRI values were calculated for each monitored site and are displayed in Figure 9.

**Figure 10:**
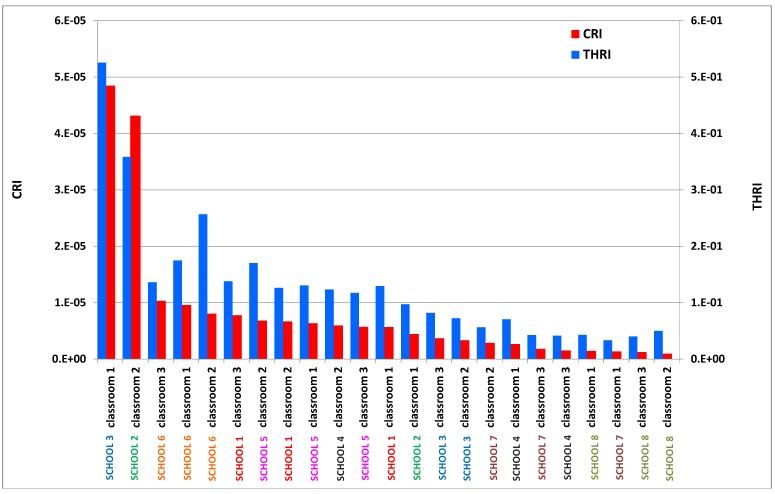
Ranking of monitored sites according to two indicators based on health risk assessment.

The indoor THRI values were higher than the corresponding outdoor values in all the monitored school buildings, with the exception of schools 4 and 8. The highest vales of THRI were found in school 2 (classroom 2) and school 3 (classroom 1). A high contribution of 1,2,3-trimethylbenzene and benzene was found in schools 2 and 3, as well as a high contribution of m,p-xylene in school 3. This finding confirms what had been found with the CRI which was that school 3 (classroom 1) and school 2 (classroom 2) should be considered environments with a high level of concern (Figure 10). This approach enabled a ranking of the investigated environments which highlighted the critical issues requiring corrective actions as well as the clean environments (schools 7 and 8) setting good practice.

## 4. Conclusions

The aim of this study was to assess the indoor air quality in the naturally ventilated school buildings by conducting a VOC monitoring campaign. The identification and quantification of VOCs and the indoor/outdoor concentration plots enabled the identification of the main emission sources. In particular, school 2 (classroom 2) and school 3 (classroom 1) presented two critical issues. At both of these sites, there were significant indoor pollutant contributions. In order to rank the sites based on their indoor air quality, two integrated indicators based on health risk assessment were used. The IAQ Cancer Risk Indicator (CRI) and the IAQ Total Hazard Ratio Indicator (THRI) enabled an assessment of the overall IAQ in the investigated sites and an estimation of the impact of the indoor activities on pupils frequenting these environments. This knowledge-based approach could be a precious tool for a more efficient management of resources with an aim of mitigating actions. Similar studies should be conducted in other schools in order to identify hazardous sources that may be contributing to poor IAQ. Furthermore, more efforts should be made to identify the VOC emission patterns of possible indoor sources. In addition, more studies are needed to evaluate whether or not there is a causal relationship between pollutant exposure and health symptoms in schools and whether this may adversely affect school performance or attendance.
